# Sensitive development windows of prenatal air pollution and cognitive functioning in preschool age Mexican children

**DOI:** 10.1097/EE9.0000000000000291

**Published:** 2024-01-09

**Authors:** Hsiao-Hsien Leon Hsu, Jamil M. Lane, Lourdes Schnaas, Brent A. Coull, Erika Osorio-Valencia, Yueh-Hsiu Mathilda Chiu, Ander Wilson, Allan C. Just, Itai Kloog, David Bellinger, Martha M. Téllez-Rojo, Robert O. Wright

**Affiliations:** a Department of Environmental Medicine and Public Health, Icahn School of Medicine at Mount Sinai, New York, New York; b Institute for Exposomic Research, Icahn School of Medicine at Mount Sinai, New York, New York; c National Institute of Perinatology, Mexico City, Mexico; d Department of Biostatistics, Harvard School of Public Health, Boston, Massachusetts; e Department of Biostatistics, Colorado State University, Fort Collins, Colorado; f Department of Geography and Environmental Development, Ben-Gurion University of the Negev, Israel; g Department of Neurology,Boston Children’s Hospital, Harvard Medical School, Boston, Massachusetts; h National Institute of Public Health, Cuernavaca, Mexico; i Department of Neurology Research, Harvard Medical School, Boston, Massachusetts

**Keywords:** Air pollution, Prenatal exposure, Particulate matter, Neurodevelopment, Sensitive windows

## Abstract

**Introduction:**

Neurotoxicity resulting from air pollution is of increasing concern. Considering exposure timing effects on neurodevelopmental impairments may be as important as the exposure dose. We used distributed lag regression to determine the sensitive windows of prenatal exposure to fine particulate matter (PM_2.5_) on children’s cognition in a birth cohort in Mexico.

**Methods:**

Analysis included 553 full-term (≥37 weeks gestation) children. Prenatal daily PM_2.5_ exposure was estimated using a validated satellite-based spatiotemporal model. McCarthy Scales of Children’s Abilities (MSCA) were used to assess children’s cognitive function at 4–5 years old (lower scores indicate poorer performance). To identify susceptibility windows, we used Bayesian distributed lag interaction models to examine associations between prenatal PM_2.5_ levels and MSCA. This allowed us to estimate vulnerable windows while testing for effect modification.

**Results:**

After adjusting for maternal age, socioeconomic status, child age, and sex, Bayesian distributed lag interaction models showed significant associations between increased PM_2.5_ levels and decreased general cognitive index scores at 31–35 gestation weeks, decreased quantitative scale scores at 30–36 weeks, decreased motor scale scores at 30–36 weeks, and decreased verbal scale scores at 37–38 weeks. Estimated cumulative effects (CE) of PM_2.5_ across pregnancy showed significant associations with general cognitive index ($\hat{CE}$ = −0.35, 95% confidence interval [CI] = −0.68, −0.01), quantitative scale ($\hat{CE}$ = −0.27, 95% CI = −0.74, −0.02), motor scale ($\hat{CE}$ = −0.25, 95% CI = −0.44, −0.05), and verbal scale ($\hat{CE}$ = −0.2, 95% CI = −0.43, −0.02). No significant sex interactions were observed.

**Conclusions:**

Prenatal exposure to PM_2.5_, particularly late pregnancy, was inversely associated with subscales of MSCA. Using data-driven methods to identify sensitive window may provide insight into the mechanisms of neurodevelopmental impairment due to pollution.

**What this study adds:**

We note that research linking prenatal ambient air pollution with children’s cognitive function has largely been using fixed clinical periods as exposure estimates such as trimesters. We linked daily prenatal PM_2.5_ exposure with advanced statistics to objectively characterize sensitive windows of childhood neurodevelopment with individual time series data. This study uses a novel advanced data-driven statistical approach developed by our team to objectively weigh the impact of PM_2.5_ on childhood neurodevelopment. Our findings suggest that prenatal exposure to PM_2.5_, particularly during late pregnancy, was inversely associated with several subscales of children’s neurodevelopment.

## Introduction

Reduced cognitive abilities and associated behavioral problems affect up to 20% of US children, potentially leading to long-term ramifications on educational attainment and career trajectories.^1–6^ Thus, it is important to understand how toxic environmental exposures during early life may alter cognitive abilities. Mounting evidence suggests that ambient air pollution, such as ambient particulate matter and traffic-related pollutants, may act as developmental neurotoxicants.^7–10^ In addition, evidence from animal, experimental, and epidemiological studies suggests that the adverse effects of air pollution on neurodevelopment likely begin in utero.^8,10^

While the mechanisms of the impact of ambient air pollution on neurodevelopment are not fully understood, we know that neurodevelopmental processes vary over life stages. Exposure to particulate matter induces oxidative stress, affecting inflammatory processes that can disrupt both organization and differentiation of the fetal brain and central nervous system (CNS)^7,11^ with effect depending in part on the timing of exposure. The fetal brain and CNS develop sequentially via an array of cell differentiation and synaptic network formation within and across multiple anatomic regions beginning during gestation in a timed cascade.^12,13^ Due to the continuous early life structural development of the fetal brain and CNS, network formation is vulnerable to environmental toxicants depending on both the dose and timing of the environmental exposure.^14^ Unlike adult exposure, which occurs in the context of a developed CNS, even slight disruption due to inflammation in early life may lead to subclinical neurotoxicity that affects later performance by altering the longer-term neurodevelopmental trajectory.^15^ The fetal brain is susceptible to air pollutants, as prior studies have demonstrated that in utero exposure during different sensitive windows (e.g., gestational trimesters) to several air pollutants, such as nitrogen dioxide (NO_2_) and PM_2.5_ levels,^16^ polycyclic aromatic hydrocarbon,^17,18^ and PM_10_^19^ increases the risk of psychomotor and mental developmental delays, lower full-scale IQ scores, and increased attention problems in infants and children.

While these studies before 2015 have linked air pollution and cognitive outcomes among children, typically, they have subjectively assigned exposure timing, most commonly assessing air pollution timing during each gestational trimester or using pregnancy-averaged air pollution exposure. For this reason, the role of exposure timing still needs to be clarified. It is difficult to compare the results between studies to define the critical windows driving fetal programming. Measuring exposure when a fetus is less susceptible to environmental stress may lead to underestimated or missed associations. However, the relevant windows are not yet defined from previous literature, and clinically defined trimesters may not necessarily correspond to fetal brain development vulnerable periods.^13^ Therefore, methods to objectively identify windows of neurotoxicant susceptibility and the impacted neurodevelopmental domains are needed to determine the underlying mechanisms and/or processes resulting in disrupted development. In recent literature, researchers from around the world have been starting to utilize these data-driven methods. However, the timing is very outcome-specific and requires more studies on each domain or executive function test to better understand the impact of prenatal air pollution.^20–22^

To better determine the association between air pollution and children’s neurocognitive development, we used daily particulate matter (diameter ≤2.5 µm; PM_2.5_) exposure data estimated over the course of gestation and applied a Bayesian distributed lag model to more precisely identify sensitive development windows of prenatal particulate air pollution exposure on a range of children’s neurodevelopmental outcomes (verbal, perceptual-performance, quantitative, memory, motor, and the general cognition) in an urban city population, which we hypothesize that the effect of prenatal air pollution occurs in a much finer temporal resolution than predefined trimesters and requires the concept of distributed lag to correctly identify this lagged association. Furthermore, these effects may occur in a sexually dimorphic manner, although the data are sparse. Therefore, to better understand the role of sex as a biological variable, we will also evaluate sex differences.

## Materials and methods

### Study population

The Programming Research in Obesity, Growth, Environment and Social Stressors study has recruited pregnant women who received prenatal care through the Mexican Social Security System (Instituto Mexicano del Seguro Social) between July 2007 and February 2011. Instituto Mexicano del Seguro Social provides healthcare to affiliated private sector employees, the majority of whom are low- to middle-income workers and their families. The recruiting eligibility criteria were women over 18 years of age with less than 20 weeks of gestation, who planned to stay in Mexico City for the next 3 years. Other criteria include access to a telephone, no major disease in medical history, and not using steroid or antiepilepsy medications or consuming alcohol on a daily basis. The recruiting procedures were approved by institutional review boards at the Harvard School of Public Health, Icahn School of Medicine at Mount Sinai, and the Mexican National Institute of Public Health. Written informed consent was provided during the visits for all participants. A total of 948 mothers giving a live birth, 571 mother-children dyads were followed longitudinally. After excluding participants who do not have a PM_2.5_ exposure estimation, our analysis included 553 full-term (≥37 weeks) children who completed McCarthy Scales of Children’s Abilities (MSCA) at the age of 4 years.

### Determination of gestational age

Ultrasounds were not routinely performed as standard of care; therefore, gestational age was based on the last menstrual period (LMP) and standardized physical examination to determine gestational age at birth.^23^ If the physical examination assessment of gestational age differed by more than 3 weeks from the gestational age determined by LMP, the physical exam was used instead of the gestational age determined by LMP.

### Prenatal PM_2.5_ prediction

**Figure 1.**
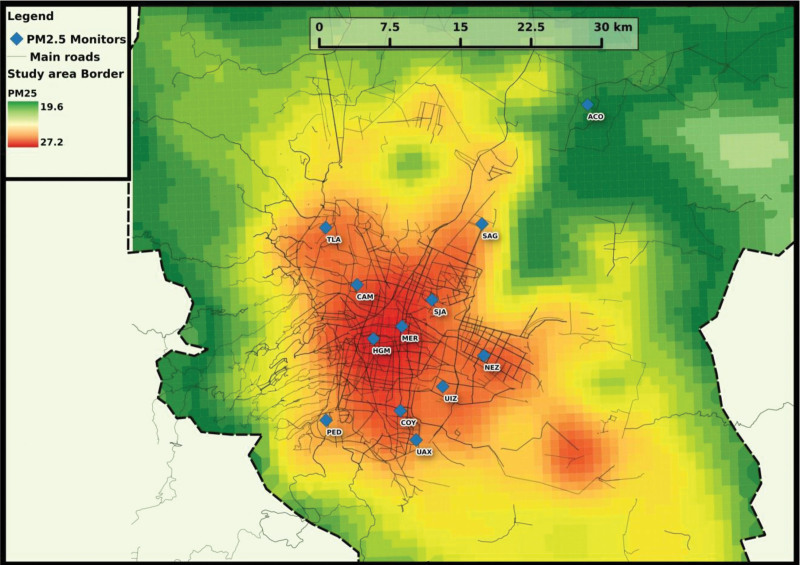
Predicted PM_2.5_ levels in Mexico City. Fine particulate matter (aerodiameter <2.5 μm; PM_2.5_) estimated by spatiotemporal land-use regression (LUR) model incorporating satellite data (Just 2014) (R^2^ = 0.74). The model utilizes moderate-resolution imaging spectroradiometer satellite-derived aerosol optical depth (AOD) measurements to derive daily values, and it incorporates day-specific calibrations of AOD data using ground PM_2.5_ measurements from 12 monitoring stations, LUR, and meteorological variables reported by the Servicio Meteorológico Nacional (SMN).

Daily PM_2.5_ levels were estimated using a validated spatiotemporal satellite-based land-use regression hybrid PM_2.5_ prediction model. Moderate-resolution imaging spectroradiometer satellite-derived aerosol optical depth (AOD) measurements were used to predict daily PM_2.5_ concentration starting in 2004 across the greater Mexico City region. We layered this remote sensing data with traditional land-use regression predictors and a spatial smoothing technique to yield residence-specific estimates of PM_2.5_ exposure. The model calibrates the relationship between satellite-measured AOD and ground-level monitor-measured PM_2.5_ daily with a grid cell of 1 × 1 km. We employed ground PM_2.5_ measurements from 12 monitoring stations maintained by the atmospheric monitoring system of the Mexico City government, which is publicly available. The density of roadways was estimated using OpenStreetMap, which was used in conjunction with meteorological variables from other local monitoring agencies, such as relative humidity, precipitation, temperature, and planetary boundary layer height. Exposure estimates were generated using linear mixed models with random slopes and intercepts to account for variation in satellite-to-ground calibration. A second model estimated exposures on days when AOD measures were not available (primarily due to cloud cover) using spatial smoothing within a season and the time-varying mean from local ground monitors. Cross-validation R^2^ from leaving out each monitor, one at a time, for prediction of daily values was 0.72 for the full model (including predictions on days without satellite data). Predicted PM_2.5_ at each participant’s residence in relation to the 1 × 1 km grid locations showed excellent heterogeneity (Figure 1). Due to Mexico’s climate, almost all participants had windows open 24 hours a day (≥94%). This removes the major concern of indoor–outdoor pollution ratio that commonly exists in studies conducted in developed countries. Although there are substantial sources of indoor air pollution, our estimates of ambient exposure levels may have more relevance for estimating the impact on health in Mexico compared with ambient exposure models in other developed countries.

### Neurodevelopment measurements—McCarthy Scales of Children’s Abilities

MSCA is a carefully constructed individual test of general cognitive ability designed to evaluate children ages 2–8 years and offers certain advantages over the Wechsler preschool and primary scale of intelligence-III and the Stanford–Binet intelligence scales at this age range.^24^ MSCA includes 18 tests that sample different functions, 15 of which are combined into a composite score known as the general cognitive index (GCI). The GCI has a standard score with a mean of 100 and a standard deviation of 16, and it includes evaluations in five domains: verbal, perceptual-performance, quantitative, memory, and motor domains. MSCA was administered at an average age of 4.7 (4.3–5.1) years. The test was conducted by pediatric neurologists at INPer (National Institute of Perinatology, Mexico City, Mexico); when the children are brought into our facility for follow-ups in this study, all tests are conducted in the same room.

### Covariates

Information about child’s sex, maternal age at birth, socioeconomic status (SES), maternal IQ (estimated using the Wechsler Abbreviated Scale of Intelligence),^25^ date of birth, and gestational age at birth was collected through direct interviews with the moms at our research facilities at the Mexican National Institute of Perinatology. Maternal blood samples were obtained during pregnancy to evaluate lead exposure. SES of participant families and information derived from prenatal questionnaire results were used to classify participants into categories based on the SES index created by the Asociación Mexicana de Agencias de Investigación de Mercados y Opinión Pública (AMAI) (Carrasco, 2002).^26^ Our study classified participants as low, medium, and high SES on a relative scale within our cohort. Women who reported smoking at enrollment were classified as prenatal smokers and were excluded from the analysis. Covariates included in the final model were child sex, child age at neurocognitive test, maternal age at delivery, SES, maternal IQ, and maternal blood lead during the second trimester. While studies show that air pollution may affect boys and girls differently when exposed during pregnancy, we have also tested using child sex as a potential effect modifier in our interaction model described below.^27^ Missing covariates are estimated by “Multivariate Imputation by Chained Equations” using “mice” packages in R (v3.5.1, Vienna, Austria).^28^

### Statistical analyses

We estimated the association between MSCA scores and weekly average maternal PM_2.5_ exposure using Bayesian distributed lag interaction models (BDLIM).^28^ BDLIM extended the traditional constrained distributed lag framework developed in time series studies^29^ and recently applied to estimate critical windows during pregnancy^28^ to account for effect modification, in our case by sex. The BLDIM for child *i* (i=1,…,n) who is sex j (j=1 for female and    j=0 for male) is


$$\begin{array}{l} E\left(Y_{i}\right)=a_{j}+\beta_{j}\overset{T}{\underset{t=1}{\sum }}w_{jt}X_{it}+Z_{i}^{\prime }\gamma , \end{array}$$

where $a_{j}$ is a fixed sex-specific intercept, $\beta_{j}$ is the regression coefficient characterizing the sex-specific association between weighted PM_2.5_ exposure and children’s MSCA score, $\overset{T}{\underset{t=1}{\sum }}w_{jt}X_{it}$ is the weighted exposure, and $Z_{i}^{\prime }\gamma$ is the covariate regression term. The $\;\;\;w_{jt}s$ identify critical windows of susceptibility, while $\beta_{j}\;$ parameterizes the within-window effect. When weights are constant, this is equivalent to using mean exposure. However, when the weights vary by time, the model assigns greater relative weight to some periods. Time periods with greater weight are critical exposure windows.

The approach considers four potential patterns of effect modification by sex by allowing $\beta_{j}$ and the weights $w_{jt}$ to be sex-specific or the same for both sexes. The four patterns are: (1) boys and girls have different critical windows (e.g., shifted by a few weeks) and the association between maternal exposure and children’s MSCA score within the window is different by sex (e.g., a stronger association for boys); (2) boys and girls have different critical windows, but the same association between exposure and MSCA score within window; (3) boys and girls have the same critical window, but a different association between PM_2.5_ and children’s MSCA score within the window; and (4) boys and girls have the same critical window and the same association between exposure and children’s MSCA score within the window (no modification). The approach quantifies the likelihood of each pattern of heterogeneity and estimates the association between exposure and outcome under the effect modification pattern best supported by the data. Hence, we can identify if the groups have the same or different critical windows and identify if the groups have the same or different within-window effects. All analysis was conducted using the “*regimes”* package in R (v3.5.1, Vienna, Austria).^28^

## Results

### Participant characteristics

Study participant characteristics are summarized in Table 1. The sample consisted of 553 boys (51%) and girls (49%) between the ages of 4 and 5 years, with a mean age of 4.7 years at the time of MSCA testing. In Programming Research in Obesity, Growth, Environment and Social Stressors, a large portion of the participants was classified as low SES. The average maternal age at birth was 32.3 (28.4–36.7) years, and the average maternal IQ was 86 (76–94). Average prenatal PM_2.5_ exposure was 22.6 (SD = 2.56) µg/m^3^ in Mexico, which is relatively high compared with average major cities in the United States and way above the 5 µg/m^3^ recommendation provided by World Health Organization. Table 1 also presents the average performance on each MSCA domain. Because we focused the analysis on full-term babies, we compared baseline characteristics between the analyzed participants and the full cohort. We did not find significant differences in basic characteristics except for gestational age.

**Table 1.** Characteristics of participating mothers and children from the Programming Research in Obesity, Growth, Environment and Social Stress (PROGRESS) study in Mexico City

	Median (IQR) or n (%)					
Characteristic	Total		Boys		Girls	
n	553		283	(51)	270	(49)
Maternal age at birth, year	32.3	(28.4–36.7)	32.7	(29–37.1)	32.1	(27.8–36.2)
Socioeconomic status (SES)						
Low	286	(52)	142	(50.4)	144	(53.5)
Medium	208	(37.7)	110	(39)	98	(36.4)
High	57	(10.3)	30	(10.6)	27	(10.1)
Maternal blood lead level during pregnancy (μg/dl)	3	(2–3.8)	3.3	(2.1–3.8)	2.8	(1.9–3.8)
Maternal IQ	86	(76–94)	86	(75–95)	86	(77–93)
Median prenatal PM_2.5_ (µg/m^3^)	23	(20.7–24.3)	23	(21–24.4)	23	(20.5–24.1)
Child age at MSCA test, year	4.7	(4.3–4.8)	4.8	(4.3–4.8)	4.7	(4.3–4.8)
McCarthy Scales of Children’s Abilities (MSCA) (scalar score)						
General cognitive index (GCI)	101	(90–99.5)	98	(89–97.9)	101	(92–101.2)
Quantitative scale	46	(40–46)	45.5	(40–45.5)	46	(40–46.5)
Memory scale	47	(41–47.1)	47	(40–46.6)	48	(42–47.7)
Motor scale	45	(38–44.3)	44	(38–43)	47	(40–45.7)
Perceptual-performance	52	(46–51.8)	51	(46–50.5)	53	(48–53.1)
Verbal scale	50	(44–50.6)	48	(43–49)	51	(44–52.3)

### Identification of critical windows using Bayesian distributed lag interaction model

**Figure 2.**
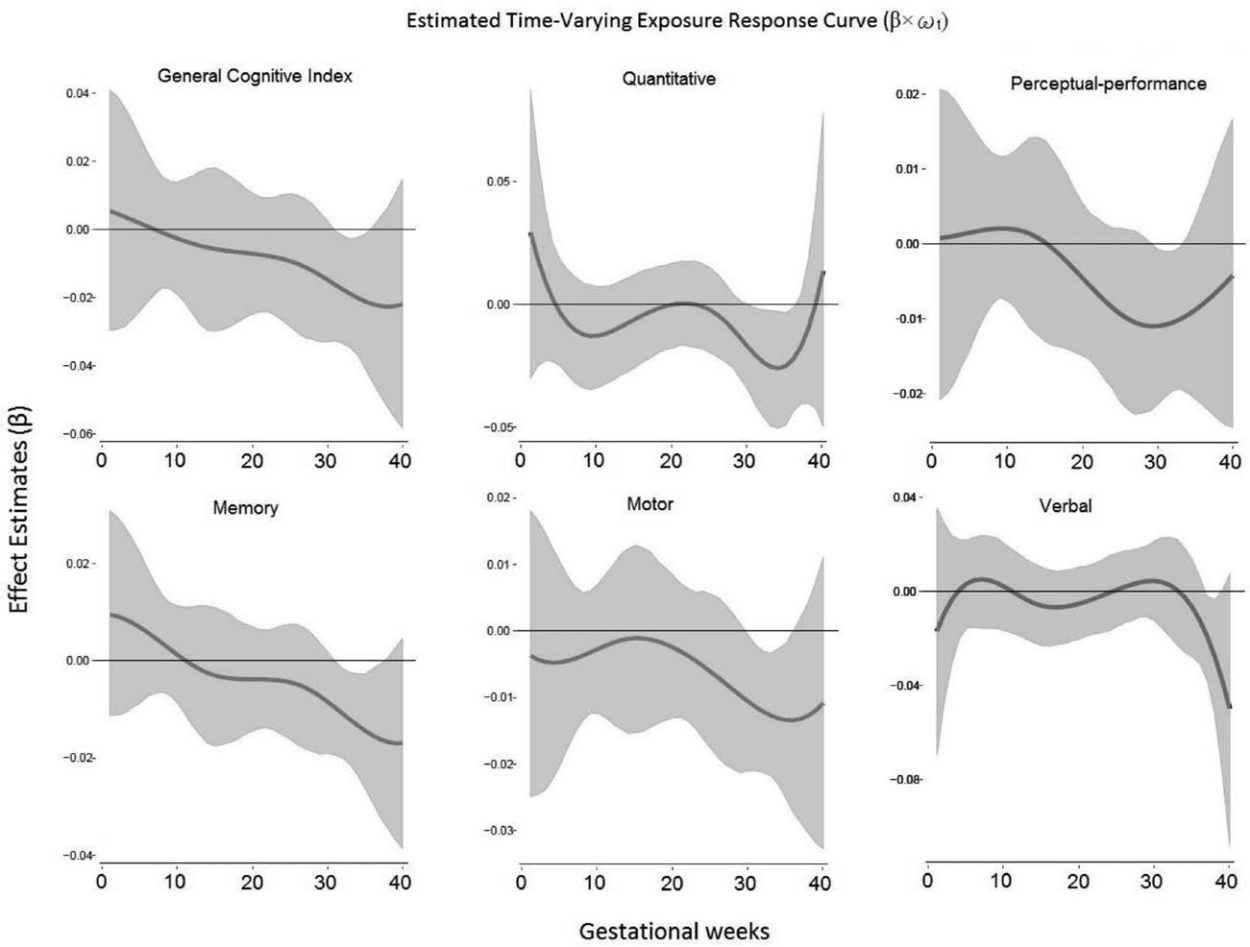
Week-specific associations between weekly prenatal PM_2.5_ levels across gestation and McCarthy Scales of Children’s Abilities (MSCA) score. Time-varying association between PM_2.5_ exposure over pregnancy and MSCA score modeled by Bayesian distributed lag interaction models. Models were adjusted for maternal age, child age, socioeconomic status, maternal IQ, and maternal blood lead; child’s sex was tested for effect estimates heterogeneity in the model and not stratified by sex. *Y* axis represents the change in MSCA scaled score associated with a 1 μg/m^3^ increase in PM_2.5_.

**Figure 3.**
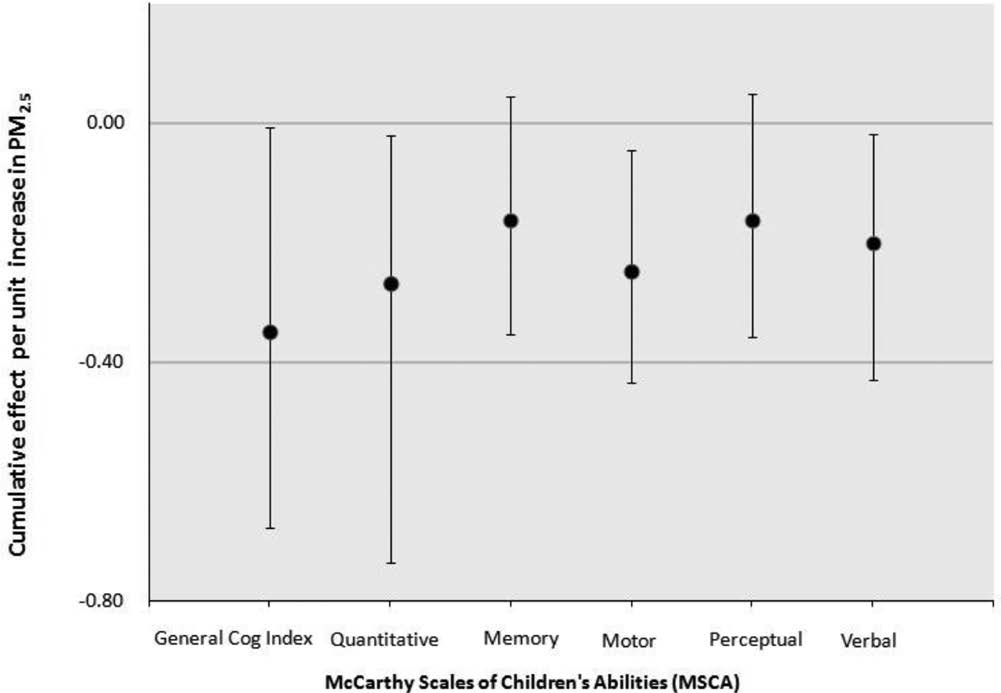
Prenatal PM_2.5_ levels across gestation and McCarthy Scales of Children’s Abilities (MSCA) score. Cumulative association and 95% confidence intervals between 1 μg/m^3^ increase in PM_2.5_ exposure over pregnancy and MSCA score modeled by Bayesian distributed lag interaction models. Models were adjusted for maternal age, child age, socioeconomic status, maternal IQ, and maternal blood lead; child’s sex was tested for effect estimates heterogeneity in the model and not stratified by sex. *Y* axis represents the change in MSCA scaled score associated with a 1 μg/m^3^ increase in PM_2.5_.

Modeled time-varying association between PM_2.5_ and MSCA outcomes are shown in Figure 2. BDLIM identified a significant association between increased PM_2.5_ exposure in late pregnancy, specifically 30–40 weeks gestation, and reduced child MSCA GCI scores and many subscales, including quantitative, motor, and verbal scales (Figure 2). Overall association showed a downward trend after conception and revealed a significant window during the weeks just before birth, suggesting that late pregnancy exposure may have a higher negative impact on the cognitive function of children. The CE is the expected difference in outcome associated with a simultaneous increase of 1 µg/m^3^ increase in PM_2.5_ in each exposure week. The estimated CE between prenatal PM_2.5_, MSCA subscale, and GCI score are adjusted for maternal age, maternal IQ, maternal blood lead, SES, child age, and sex are shown in Figure 3 and Table 2. PM_2.5_ had the following estimated CEs across pregnancy: GCI ($\hat{CE}$ = −0.35; 95% CI = −0.68, −0.01; critical windows: 31–35 weeks; *P* = 0.025), quantitative scale ($\hat{CE}$ = −0.27; 95% CI = −0.74, −0.02; critical windows: 30–36 weeks; *P* = 0.01), memory scale ($\hat{CE}$ = −0.16; 95% CI = −0.35, 0.04; critical windows: 31–37 weeks; *P* = 0.05), motor scale ($\hat{CE}$ = −0.25, 95% CI = −0.44, −0.05; critical windows: 30–36 weeks; *P* = 0.004), perceptual-performance scale ($\hat{CE}$ = −0.16; 95% CI = −0.36, 0.05; critical windows: 30–33 weeks; *P* = 0.059), and verbal scale ($\hat{CE}$ = −0.2; 95% CI = −0.43, −0.02; critical windows: 37–38 weeks; *P* = 0.015). Although the CE of memory and perceptual-performance scales was marginally statistically significant when deriving the confidence interval summarizing the effect of entire prenatal PM_2.5_ exposure, significant critical windows were identified by the model showing a significant negative correlation between prenatal PM_2.5_ exposure and MSCA subscales.

**Table 2.** BDLIM model summary showing unweighted effect estimates and cumulative effect estimate for the duration of pregnancy

	Effect estimates (β)			Cumulative effect estimates(β × ωt)				
	Mean	Low	High	Mean	Low	High	Pr>0	Windows (weeks)
General cognitive index	−0.02	−0.03	0.00	−0.35	−0.68	−0.01	0.02	31–35
Quantitative scale	−0.02	−0.03	−0.01	−0.27	−0.74	−0.02	0.01	30–36
Memory scale	−0.01	−0.02	0.01	−0.16	−0.35	0.04	0.05	31–37
Motor scale	−0.01	−0.02	−0.01	−0.25	−0.44	−0.05	0.00	30–36
Perceptual-performance	−0.01	−0.01	0.01	−0.16	−0.36	0.05	0.06	30–33
Verbal scale	−0.02	−0.03	−0.01	−0.20	−0.43	−0.02	0.01	37–38

All models adjusted for maternal age, child age at cognitive testing, socioeconomic status, maternal IQ, and maternal blood lead; child’s sex was tested for effect estimates heterogeneity in the model and not stratified by sex.

BDLIM indicates Bayesian distributed lag interaction models.

Effect modification by child sex was examined in all models. However, based on posterior model probability and deviance information criterion, our models indicate that there were no sex-based interactions detected across our analysis.

## Discussion

In the current study, we investigated the effects of prenatal exposure to fine PM_2.5_ on children’s general cognitive development at age 4–5 years. To investigate these effects, we applied a distributed lag regression (e.g., BDLIM) to determine the time boundaries of sensitive windows. Our study findings show that prenatal maternal PM_2.5_ exposure is adversely associated with cognitive abilities in young children, and the critical windows of susceptibility occurred in late pregnancy. We did not observe a significant difference in the effects of exposure depending on children’s biological sex.

We leveraged high-resolution daily residential estimated PM_2.5_ exposure estimates and BDLIM to objectively modeling the potential windows of neurodevelopmental vulnerability to avoid subjectivity created by measuring predetermined integrated environmental exposure levels. The advantage of our data-driven statistical approach is the ability to identify sensitive windows for PM_2.5_ effects on neurodevelopment more precisely. Previous studies suggest that modeling effect estimates using PM_2.5_ averaged over clinically defined trimesters can be subject to bias^28^ due to the seasonal PM_2.5_ pattern and correlation structure. In addition to identifying the window when the exposure–outcome correlation was strongest, implementing BDLIM also estimated unbiased association across other weeks during pregnancy. The modeling approach also allows us to identify associations, even with a narrow sensitive window or across different trimesters.

Our findings are consistent with previous studies, showing that the neurotoxic effects of prenatal air pollution can adversely impact fetal neurodevelopmental functions. Specifically, animal studies show that particulate air pollution induces neuro-inflammatory processes in offspring, which may subsequently disrupt neurodevelopment.^30–33^ Further, rodent studies demonstrate that increased neuro-inflammation induced by particulate matter exposure is linked to structural changes in the brain, with smaller brain volume and reduced thickness of the prefrontal cortex,^34^ increasing the risks of neurodevelopment impairment. In a recent European study, Guxen et al^35^ also observed thinner cortex in humans (mean fine particle levels were 20.2 μg/m^3^ [range, 16.8–28.1 μg/m^3^]). These structural changes, in turn, are linked to attention deficit disorders in humans.^36^

While not being able to directly study the mechanisms, based on our results and the significant association in the exposure timing we suggest four possible mechanisms for our findings, all of which are prominent in the third-trimester window—we found for PM_2.5_. The first is hippocampal development, with some human research showing that hippocampal development parallels the third trimester^37,38^ window found in our study and could underlie our findings on cognition, as measured by McCarthy cognitive tests. It is important to note that cognitive abilities, especially memory, are regulated in the hippocampus. Any disruption in the hippocampal development during the third trimester can be consistent with our findings. By 32–34 weeks of gestational age, hippocampal neurons undergo a period of rapid enlargement and morphologic maturation.^39,40^ This sensitive period of rapid brain growth and development may make the fetal brain susceptible to even subtle toxic environmental exposures such as air pollution. Prenatal exposure to air pollution may induce long-term functional effects because it offsets the trajectory of subsequent developmental periods and thus can be of particular importance—a concept consistent with the developmental origins of health and disease theory.^41^ In addition, the dentate gyrus, which is proximal to the hippocampus and its subregion, is one of the last brain regions to develop. It only assumes a mature cytoarchitecture after 34 weeks of gestational age, according to the study of Amaral et al.^42^ Thus, the dentate gyrus is also vulnerable to exposure to air pollution during the late stages of prenatal development. This could also potentially contribute to the adverse association between prenatal PM_2.5_ exposure and neurodevelopment.

The second possible mechanism is the interference with myelination. Myelin is produced by Schwann cells, which envelope neurons, producing an insulation effect that promotes more rapid conduction of neuronal transmissions. The maturation of a layer of insulating myelin that forms around axons to allow nerve impulses to travel faster—begins at approximately 20 weeks gestation and is complete at roughly 28–30 weeks gestation.^13^ After the maturation of nerve cells, the fetal brain rapidly grows in the last 13 weeks of gestation, often tripling the weight of the brain. Notably, our analysis suggests that cognitive function strongly relates negatively with mid–late pregnancy PM_2.5_ exposure.

The third is neuro-apoptosis, a normal process of pruning damaged cells or cells no longer needed in development. Evidence suggests that neuro-inflammation induced by air pollution may also disturb normal programmed cell death, a cellular mechanism called neuro-apoptosis, which is part of normal neural differentiation and occurs around mid-to-late pregnancy in multiple brain regions.^13^ Our findings of exposure to air pollution during this period might have an impact on this process. Although disruption from air pollution is continuous throughout gestation, the strongest associations were observed for exposure occurring during mid–late pregnancy, concurrent with the most rapid fetal brain growth, myelination, and the end phase of neuronal differentiation through neuro-apoptosis.

Previous studies have demonstrated that the hippocampus affects memory functions depending on appropriate neuro-apoptosis.^34,43^ The growth and development of neuro-apoptosis in the CA1–3 fields of the hippocampus, and the proliferation of granule neurons inside the hippocampus, may be hampered by the neuro-inflammation brought on by air pollution.^44^ The hippocampus and its associated brain-derived neurotrophic factor levels, which are necessary for memory-related plasticity processes at hippocampal synapses, may also be dysregulated by inflammatory reactions in the brain.^45^ Further, evidence suggests that several cellular mechanisms of neuro-inflammation induced by particulate air pollution may drive reduced cognitive abilities. Animal studies suggest particulate air pollution exposure activates microglia,^46^ immune cells of the CNS essential in synaptic plasticity, neural development, neural formation, and maturation, which may become a chronic source of brain inflammatory factors. The human brain naturally regulates how microglia interact with neurons to form synaptic networks. However, exposure to air pollution or other environmental stressors may cause neuro-inflammation and undesirably affect this regulating mechanism, further driving the dysregulation of the normal neuron-glia interaction, with abnormal glial reaction subclinically affecting neurodevelopment.^47^ Neuro-inflammation during mid–late pregnancy is not anatomically specific in a certain brain region. However, a fourth possible mechanism is that it may associate with a disrupted ventral tegmental area (VTA) development, which is the site of dopaminergic neuron cell bodies that project to the frontal and prefrontal cortex^48,49^ via challenged neuro-apoptosis process, myelination, and synaptic pruning and maturation. VTA is critical to problem-solving, attention, and motivation behavior. More studies are needed to determine the role of exposure timing and disruption of neuro-inflammation, hippocampus and VTA development, and microglial activation.

In addition, prior animal and human evidence suggest sex-specific differences in the association between prenatal air pollution and fetal neurodevelopment.^31,50^ Studies show that air pollution may affect boys and girls differently when exposed during pregnancy. For example, Chiu et al^27^ investigated the sexually dimorphic association between various cognitive batteries (i.e., WRAML II, Conner’s CPT, and WISC-IV) and air pollution in children, showing differences in attention and memory domains. However, testing in this study was performed at a later stage (age 6–7) compared with the current study’s age range (age 4–5). In a previous study conducted by our team, we found that particulate air pollution exposure on cognitive functioning among US children occurs in a sexually dimorphic manner.^51^ However, in our current analysis with a Mexican cohort, we did not find evidence that associations with prenatal air pollution exposure on neurodevelopment are stronger in one sex compared with the other. We hypothesize that potential sex differences may not have yet manifested in children during early stages. A potential reason may include the higher-order nature of McCarthy scales, which integrate multiple cognitive functional domains. Therefore, it possibly limits the ability to detect sex-specific differences, which may even average out in a nonspecific test of cognition such as IQ. Also, a previous study found that the attention domain tends to have greater observed gender differences, which MSCA does not assess directly.^27^ Another potential reason could be that particulate air pollution levels in this Mexican cohort are two-fold higher than studies conducted in the United States. Further, indicating that the effect of the exposure could overcome human sexual dimorphism on the defense mechanism against particulate air pollution and the sexual difference in susceptibility can only be observed when exposed to a lower dose of PM_2.5_. More studies are needed to examine sex-specific associations in this context to determine whether and when sex differences may play a role in determining the impact of air pollution exposure on fetal neurodevelopment.

### Strength and limitations

A strength of our study is that the study population is located in an urban area with higher air pollution exposure on average. However, the exposure range is also much more expansive than most US cities, allowing us to see an exposure gradient. This allows us greater power to examine the effect of ambient air pollution on neurocognitive outcomes. Another strength is temporal resolution, as we generate high temporal resolution of PM estimates for each participant across gestation using a validated hybrid spatiotemporal satellite-based model.^52^ Because of our spatiotemporal resolution, we could leverage these exposure estimates to implement a data-driven advanced statistical method to identify the age boundaries of susceptibility windows for PM_2.5_ objectively. Moreover, our SES is a multivariable derived indicator variable explicitly developed for Mexico and is used in many other studies,^53^ which is a strength.

Some limitations of our study are that while we were able to control for several factors important in child neurodevelopment, such as postnatal air pollution exposure, SES, and maternal blood lead, we have limited data on factors temporally co-varying with PM exposure, such as other air pollutants (e.g., NO_2_ O_3_, etc.), and environmental noise. In addition, we did not have detailed information about household cooking and ventilation to account for indoor PM_2.5_ sources. Our study is also more generalizable to full-term children, due to our analysis excluding preterm children. Our results may be most generalizable to urban Latin American populations, but they should also be relevant to other populations.

## Conclusions

We identified the third trimester as a susceptibility window for the relationship between prenatal PM_2.5_ exposure and general neurodevelopmental child outcomes in children. Our findings suggest that increased exposure to prenatal particulate air pollution may not have sex-specific effects at age 4–5, which may be due to this young age, long before the onset of puberty. Combining data-driven statistical modeling methods with high-resolution spatial-temporal exposure modeling prediction allows us to identify the exposure timing windows in which the effects of pollution are most associated with the outcomes of interest. Such information may help identify the most vulnerable life stages (i.e., late pregnancy) and inform the optimal timing of intervention measures. Furthermore, further research in specific domains (e.g., behavior, mental health, and motor) is needed and should address exposure timing and collaborate with in vivo animal research to elucidate further possible biological mechanisms driving neurodevelopmental toxicity. The identified sensitive windows likely reflect a specific biological pathway within corresponding brain/nervous system regions, but suggest that across all test domains, mid–late pregnancy has the maximum impact on children’s cognitive performance at the age of 4 years. Our findings draw attention to the importance of timing when linking exposures and outcomes and inform the design of future studies. Lastly, we show that a more refined determination of the time window in which PM_2.5_ has the greatest impact on fetal neurodevelopment may enhance understanding of the neurotoxic effects of air pollution.
